# Intraspecies differences in natural susceptibility to amphotericine B of clinical isolates of *Leishmania* subgenus *Viannia*

**DOI:** 10.1371/journal.pone.0196247

**Published:** 2018-04-26

**Authors:** Carlos Franco-Muñoz, Merab Manjarrés-Estremor, Clemencia Ovalle-Bracho

**Affiliations:** Hospital Universitario Centro Dermatológico Federico Lleras Acosta E.S.E., Bogotá D.C., Colombia; National Centre For Cell Science, INDIA

## Abstract

Amphotericin B (AmB) is a recommended medication for the treatment of cutaneous and mucosal leishmaniasis in cases of therapeutic failure with first-line medications; however, little is known about the *in vitro* susceptibility to AmB of clinical isolates of the subgenus *Viannia*, which is most prevalent in South America. This work aimed to determine the *in vitro* susceptibility profiles to AmB of clinical isolates of the species *L*. *(V*.*) panamensis*, *L*. *(V*.*) guyanensis and L*. *(V*.*) braziliensis*. *In vitro* susceptibility to AmB was evaluated for 65 isolates. Macrophages derived from the U937 cell line were infected with promastigotes and exposed to different AmB concentrations. After 96 hours, the number of intracellular amastigotes was quantified by qPCR, and median effective concentration (EC_50_) was determined using the PROBIT model. The controls included sensitive strains and experimentally derived less sensitive strains generated *in vitro*, which presented EC_50_ values up to 7.57-fold higher than the values of the sensitive strains. The isolates were classified into groups according to their *in vitro* susceptibility profiles using Ward’s hierarchical method. The susceptibility to AmB differed in an intraspecies-specific manner as follows: 28.21% (11/39) of *L*. *(V*.*) panamensis* strains, 50% (3/6) of *L*. *(V*.*) guyanensis* strains and 34.61% (9/26) of *L*. *(V*.*) braziliensis* strains were classified as less sensitive. The latter subset featured three susceptibility groups. We identified Colombian isolates with different AmB susceptibility profiles. In addition, the capacity of species of subgenus *Viannia* to develop lower susceptibility to AmB was demonstrated *in vitro*. These new findings should be considered in the pharmacovigilance of AmB in Colombia and South America.

## Introduction

Leishmaniasis is a group of vector-transmitted diseases of zoonotic origin caused by infection with different species of *Leishmania*, which is a protozoan parasite of family *Trypanosomatidae* (order Kinetoplastida). These parasites are transmitted to a mammal through the bites of hematophagous dipteran insects belonging to genus *Phlebotomus* in the Old World and genus *Lutzomyia* in the New World [1]. This disease is present in 98 countries in the tropics, subtropics and southern Europe, and an estimated 350 million people are at risk of infection. Between 700,000 and 1.2 million cases of cutaneous leishmaniasis and between 200,000 and 400,000 cases of visceral leishmaniasis are estimated to occur each year, resulting in between 20,000 and 40,000 deaths per year due to this disease [1].

In the Americas, leishmaniasis constitutes a public health problem due to its high incidence, morbidity, broad geographical distribution, parasite species variety and clinical forms. In the Americas, 15 of the 22 *Leishmania* species that can cause disease in humans have been identified. In addition, reports have identified 54 vector species that are potentially involved in parasite transmission [2].

One of the main challenges regarding leishmaniasis is related to treatment, which involves the administration of toxic and poorly tolerated medications [3]. A limited number of drugs are available for the treatment of leishmaniasis. The most commonly used medications are pentavalent antimony salts, such as meglumine antimony (Glucantime^®^) and sodium stibogluconate (Pentostam^®^). Other medications in use are miltefosine, pentamidine, aminosidine, aminoglycosides, pentoxifylline, azole derivatives and amphotericin B (AmB) [1, 4, 5]. The effectiveness of medications is influenced by several factors, including pharmacokinetics, pharmacogenetics, pharmacodynamics, host immune responses, the clinical forms and specie’s intrinsic or acquired susceptibility differences to the drug [6, 7].

Controlled clinical trials have been conducted for several of the medications in use [8, 9]; however, there are no reports of this type of study with AmB for the treatment of cutaneous and mucosal leishmaniasis. This medicine is recommended as the second line of treatment for these clinical forms of the disease in cases of therapeutic failure with pentavalent antimonials [2].

Although a controlled clinical trial that has appropriately evaluated the utility of AmB for the treatment of cutaneous and mucosal leishmaniasis has not been reported [8–10], various case reports with cure rates between 90 and 100% have been registered [11–14]. However, cases of therapeutic failure have been reported in India [15] and France [16], and cases of relapse have been reported in the Americas [11, 17, 18]. The cause of treatment failure is not easily established in patients with leishmaniasis due to the multifactorial nature of the disease. The intrinsic or acquired susceptibility of the parasite species can contribute to this outcome. In the case of AmB, although multiple reports have investigated the *in vitro* susceptibility of clinical isolates and reference strains to the drug, these works have tended to focus on Old World species, and few reports have investigated clinical isolates of subgenus *Viannia* [19, 20]. In South America, the most prevalent species of *Leishmania* correspond to this subgenus [21, 22]; however, only three studies that have reported on susceptibility to AmB included clinical isolates of *L*. *(V*.*) braziliensis* and *L*. *(V*.*) guyanensis*. These studies used promastigotes with variable drug exposure times [23] [24].

In Colombia, the baseline susceptibility to AmB is unknown; however, reports from other countries and *in vitro* experiments with other drugs suggest that these species have the ability to introduce changes in their susceptibility profiles to anti-leishmania drugs and that intra- and interspecies differences in susceptibility may exist [19,25, 26]. Moreover, strains of *L*. *(L*.*) mexicana*, *L*. *(L*.*) donovani* and *L*. *(L*.*) tarentolae* resistant to AmB have been generated *in vitro* by increasing concentrations of the drug [27–30].

Considering the above findings, this work aimed to evaluate the patterns of *in vitro* susceptibility to AmB in clinical isolates and reference strains of species of subgenus *Viannia* and to determine the baseline susceptibility to this compound for isolates of *L*. *(V*.*) braziliensis*, *L*. *(V*.*) panamensis* and *L*. *(V*.*) guyanensis*.

## Materials and methods

### Study population

We included 65 clinical isolates of the *species L*. *(V*.*) panamensis*, *L*. *(V*.*) braziliensis* and *L*. *(V*.*) guyanensis* from patients treated at Hospital Universitario Centro Dermatológico Federico Lleras Acosta, Bogotá DC, Colombia, which is a national reference center for leishmaniasis. All of the strains were isolated from patients with cutaneous leishmaniasis without prior treatment from the five natural regions of the country. The isolates were collected between 2000 and 2015 and were selected randomly from the center’s biological bank. The identification of species was performed by using PCR-RFLP profiling of the hsp70 and miniexon gene according to the reported methodology [31, 32].

The sample size was calculated based on the formula for the estimation of proportions for a sample compared to a hypothetical value. The STATA SE version 13 software was used. The number of isolates per species was determined based on the relative prevalence of the species in Colombia [21]. A total of 37 isolates were analyzed for *L*. *(V*.*) panamensis*, 24 for *L*. *(V*.*) braziliensis* and 4 for *L*. *(V*.*) guyanensis*.

### Ethical aspects

This study was approved and monitored by the Ethics Committee of the Hospital Universitario Centro Dermatológico Federico Lleras Acosta in accordance with national regulations and the Declaration of Helsinki. The patients signed an informed consent statement for the use of their clinical information and *Leishmania* isolates. The included samples were anonymized to ensure that identification of the patient was not possible.

### Drugs

Amphotericin B deoxycholate solution A2342 (Sigma-Aldrich).

### Controls: Sensitive strains and strains with decreased in vitro susceptibility to AmB

Internal standards consisting of the sensitive strains and experimentally derived less sensitive strains were included. The sensitive strains corresponded to promastigotes of *Leishmania (V*.*) panamensis* (MHOM/PA/71/LS94), *Leishmania (V*.*) braziliensis* (MHOM/BR/00/M2903) and *Leishmania (V*.*) guyanensis* (MHOM/GF/79/LEM85) acquired from the *Center National De Reference Des Leishmania* (Montpellier, France).

To generate the less sensitive strains, clones derived from the reference strains were obtained by the limiting dilution method according to the reported methodology [33]. The promastigotes were grown to an initial concentration of 1x10^6^ parasites/ml in Schneider medium (Sigma-Aldrich) supplemented with 10% fetal bovine serum (FBS) at 26°C for six days until they reached stationary phase. Subsequently, the parasite concentration was adjusted to 1x10^4^ parasites/ml, and serial dilutions were made at a 1:2 ratio in 96-well plates until wells with a single parasite were obtained.

One clonal line generated for each reference strain was selected and subjected to increasing concentrations of AmB, starting with a concentration of 0.01 μg/ml (Sigma-Aldrich) and increasing gradually to a final concentration of 0.5 μg/ml. The culture medium was changed every 48 hours. The parasites able to grow in the presence of the medication at the highest concentration were considered less sensitive and were used as controls in the *in vitro* infection assays. The maximum amphotericin B deoxycholate concentration used in the experiment was 8 times higher than the maximum concentration (*Cmax*) of free AmB in serum reported in the literature (0.06 μg/ml) [34].

### Susceptibility of promastigotes of the control strains

The parasites were grown at a concentration of 1x10^6^ parasites/ml in Schneider medium (Sigma-Aldrich) supplemented with 10% FBS at 26°C and exposed to different AmB concentrations (between 0.01 and 1 μg/ml). After 48 hours, viability was evaluated by staining with 500 nM propidium iodide (Invitrogen) and counting under a microscope. All the experiments were performed in triplicate.

### In vitro susceptibility assays for AmB with intracellular amastigotes

Tests were conducted to evaluate the susceptibility of the sensitive and less sensitive control strains and the clinical isolates to AmB. Susceptibility was determined based on the reduction in the number of intracellular amastigotes in macrophages derived from the monocyte cell line U937 (American Type Culture Collection, CRL-1593.2, USA) [26, 35]. Differentiation of U937 cells into macrophages was induced by incubating 1.2 x 10^5^ cells for 120 hours with 100 ng/ml forbol-12-myristate-13-acetate (PMA) in RPMI 1640 medium (Sigma-Aldrich) supplemented with 10% FBS at 37°C in a 5% CO_2_ atmosphere on a glass matrix in a 24-well plate.

Promastigotes in stationary phase were used for the *in vitro* infection of macrophages. The parasites were quantified and opsonized with human AB serum for one hour at 34°C. Then, the concentration was adjusted to 1.2 x10^6^ parasites/ml to obtain a 1:10 (macrophage:parasite) multiplicity of infection.

Parasites suspended in 1 ml of medium were added to each well contained macrophages. The plate was incubated for two hours at 34°C, subsequently washed three times with 34°C preheated phosphate-buffered saline (PBS, pH 7.2) to remove extracellular parasites. Finally, RPMI medium supplemented with 10% FBS was added, and the plates were incubated at 34°C in a 5% CO_2_ atmosphere. One well of the plate was used as a control to evaluate the percentage of initial infection after 24 hours by Giemsa staining and evaluating the cells under a microscope. The experiment was continued only if an infection percentage greater than 70% of the cells was obtained. The culture medium was replaced with RPMI 1640 medium supplemented with 10% FBS and different concentrations of AmB (0.02, 0.06, 0.17 and 0.5 μg/ml). All experiments included three replicates for each isolate and control strain. Infected macrophages were cultured in presence of AmB for 96 hours, the culture medium was changed at 48 hours to avoid loss of activity of the compound.

At the end of the experiment, the culture medium was removed, and proteinase K was added. The plate was incubated at 56°C overnight with shaking. Then, DNA was extracted from the cells using the DNeasy Blood & Tissue commercial extraction kit (Qiagen^®^). DNA samples were used to determine the number of amastigotes.

### qPCR to estimate the number of parasites

To quantify the number of amastigotes in infected macrophages after drug exposure, a 226-bp fragment of the miniexon gene was amplified using the primers described by Marfurt in 2003 [36]. The Bio-Rad C1000 thermocycler was used for the qPCR. The reaction mixture contained 500 nM of each primer, 7.5 μl of the SsoFast^™^ EvaGreen^®^ blend Supermix (Bio-Rad), 1 μl of DNA from infected macrophages and water to a final volume of 15 μl. The thermal profile used was an initial cycle of denaturation at 95°C for 3 min, followed by 40 cycles of 95°C for 30 s and annealing and extension at 60°C for 1 min.

To determine the number of parasites in each sample, a standard curve was constructed from known concentrations of the pGEM-T easy plasmid (Promega) ligated to a 226-bp fragment of the miniexon gene previously amplified by PCR. The number of copies of the plasmid per ng of DNA was determined based on the size of the plasmid plus the size of the amplified fragment for a total of 3243 bp. Serial dilutions of plasmid DNA were made to create a standard curve (between 1.68 x 10^9^ and 1.68 x 10^3^ copies of the plasmid). The quality parameters of the curve, such as the PCR efficiency, linear range and correlation coefficient, were obtained using the CFX manager software from Bio-Rad. To estimate the equivalent number of parasites from the number of copies of the plasmid, the approximate value of the number of copies of the miniexon gene in the genome of *Leishmania spp* (200 copies per genome) was used as a reference [37–39].

### Determination of the median effective concentration (EC_50_) of amphotericin B and calculation of the reduction percentage of the number of parasites

The median effective concentration (EC_50_) values were calculated using the PROBIT procedure of the IBM SPSS program version 20.0 as previously reported [35]. The percent reduction of number of parasites was determined for the clinical isolates and reference strains at a concentration of 0.5 μg/ml AmB, and the distribution of this percent reduction was described for each species. This concentration was selected because it was 8 times higher than the reported C_max_ of free AmB and was the highest concentration used during the generation of the least sensitive control strain.

To evaluate the differences in the EC_50_ values, and in the percent reduction between the control strains and clinical isolates by species, Kruskal-Wallis nonparametric analysis of variance was used, followed by Dunn’s multiple comparisons test when more than two groups were compared.

### Classification of clinical isolates by susceptibility

To classify the clinical isolates into different groups according to the degree of *in vitro* susceptibility to AmB, Ward’s method of hierarchical classification was used to separate the strains into two groups, based on minimizing the total within-cluster variance [40, 41]. The clusters were classified using the variables EC_50_ and percentage of reduction observed in the strains for each species. The results were shown in a dendrogram (S1 Fig). The categories of susceptible and less susceptible strains were denoted when two clusters were identified in the species, while in the species that were observed three clusters the category of more susceptible strains was additionally denoted. Comparison of mean ranges to evaluate the intra-species differences hypothesis for each degree of susceptibility between the groups were performed using the Mann-Whitney U test and the Kruskal-Wallis one-way analysis of variance followed by Dunn’s multiple comparison test as required. All statistical tests were performed in STATA SE software version 13 with a 5% level of significance. Graphs were constructed in GraphPad Prism software version 7.0.

## Results

### Generation of control strains with decreased in vitro susceptibility to AmB

**Fig 1:**
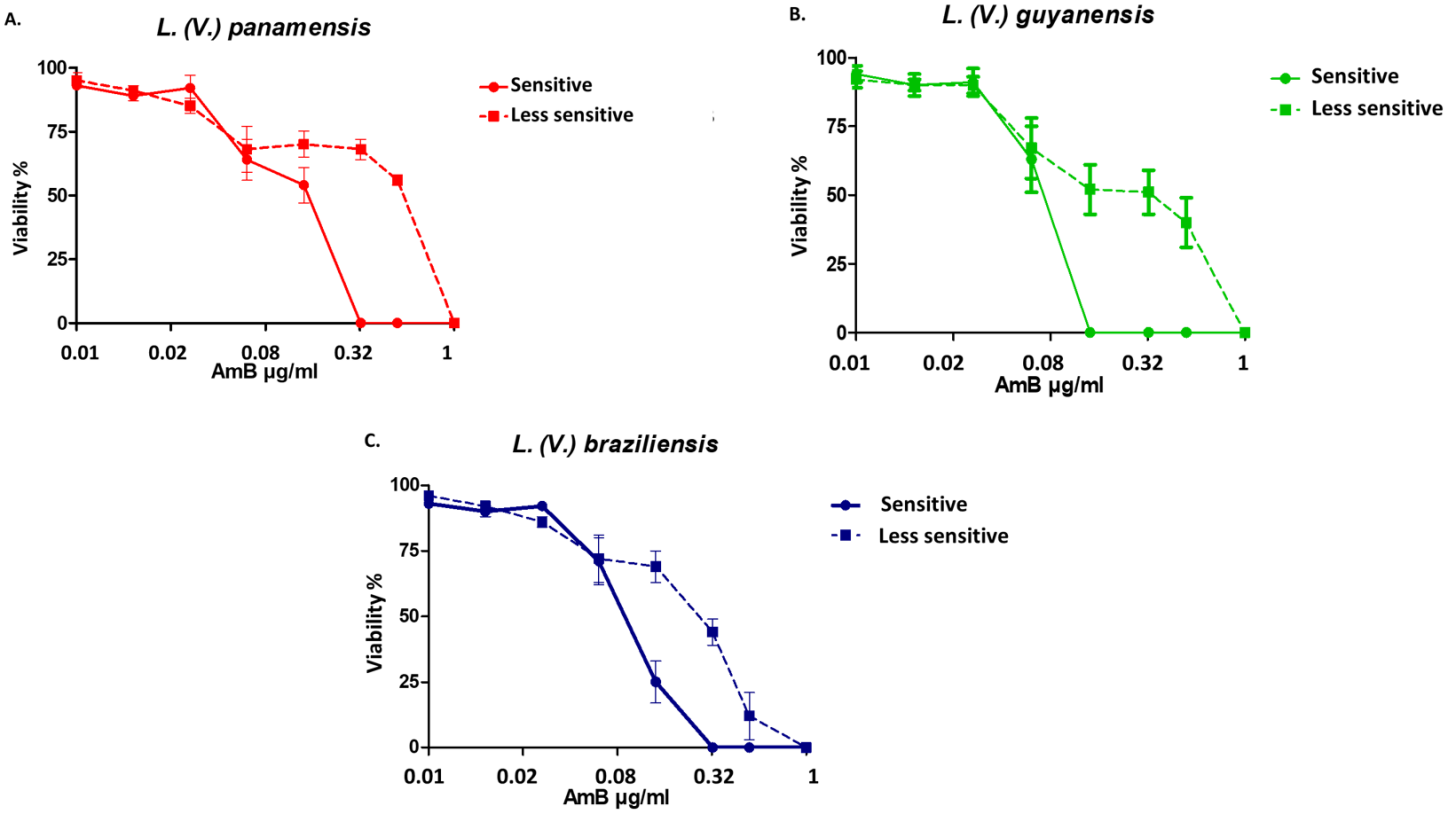
Percentage of viability of promastigotes to different concentrations of AmB. Promastigotes of the reference strain for each species were selected by gradually increasing the AmB concentration. The dotted line graphs indicate the less sensitive promastigotes. The *y-*axis shows the percentage of viability, and the different AmB concentrations used are shown on the *x-*axis. The experiments were performed in triplicate.

Less sensitive promastigotes were obtained from all species After 32 rounds of culture with gradual increases in the AmB concentration. The sensitive promastigotes exhibited 100% viability reduction following exposure of *L*. *(V*.*) panamensis* and *L*. *(V*.*) braziliensis* to 0.32 μg/ml AmB and *L*. *(V*.*) guyanensis* to 0.16 μg/ml. In contrast, a concentration of 1 μg/ml AmB was necessary to show an effect on viability for the less sensitive strains of the three species (Fig 1). These results suggest that the strains generated *in vitro* present differences in their susceptibility to AmB in the promastigote stage.

### EC_50_ and percent reduction of intracellular parasites of the control strains

**Fig 2:**
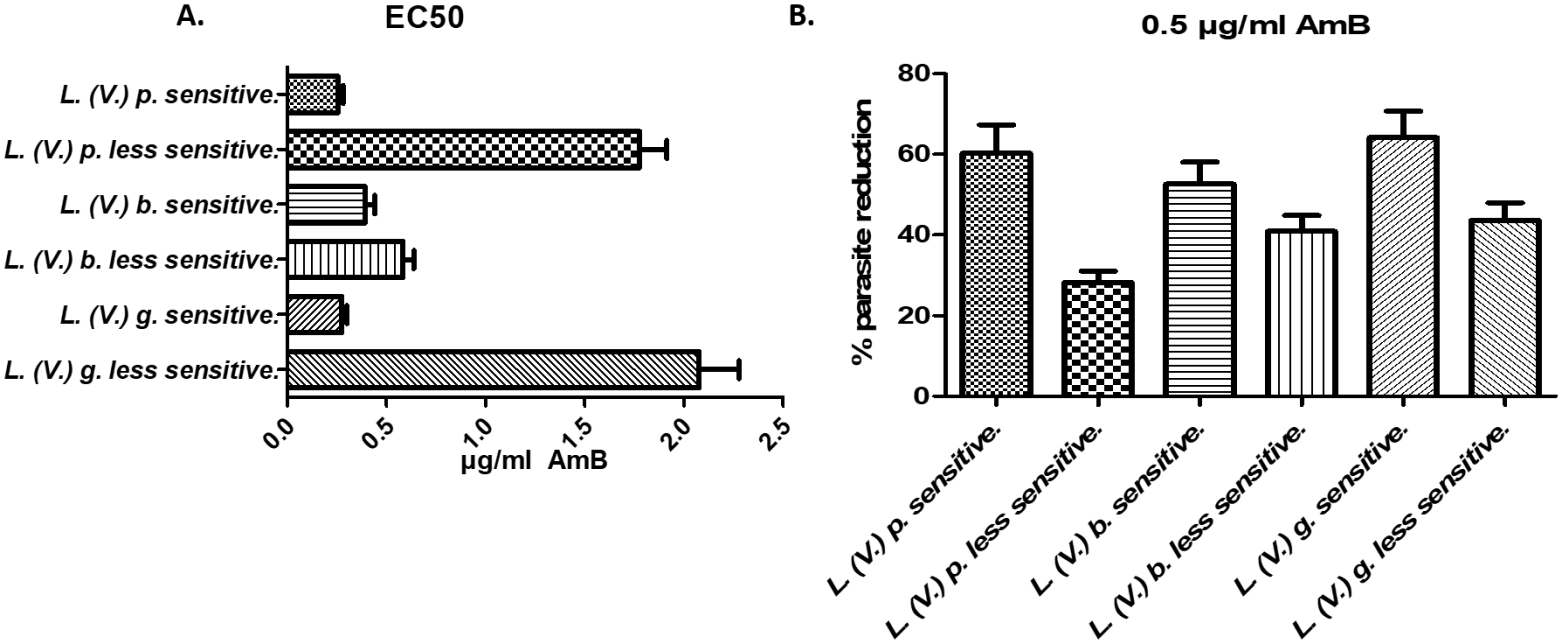
*In vitro* susceptibility to AmB of intracellular amastigotes of the sensitive control strains and less sensitive strains generated *in vitro*. A) EC_50_ values for AmB calculated for intracellular amastigotes of the less sensitive and sensitive strains. B) Percent reductions in the number of parasites of the sensitive control strains and less sensitive strains generated *in vitro* after exposure of infected macrophages to 0.5 μg/ml AmB. *L*. *(V*.*) p*. *less s*: *L*. *(V*.*) panamensis less sensitive*. *L*. *(V*.*) b*. *s*: *L*. *(V*.*) braziliensis sensitive*. *L*. *(V*.*) b*. *less s*: *L*. *(V*.*) braziliensis less sensitive*. *L*. *(V*.*) g*. *s*: *L*. *(V*.*) guyanensis sensitive*. *L*. *(V*.*) g*. *less s*: *L*. *(V*.*) guyanensis less sensitive*. The experiments were performed in triplicate.

Significant differences in the EC_50_ values and percent parasite reductions were found (p<0.05) between the sensitive and less sensitive strains for each species (Fig 2A and 2B). The specie that showed the greatest change was *L*. *(V*.*) guyanensis* less sensitive strain that exhibited a 7.57-fold increase in the EC50 with respect to the susceptible control strain. For the species *L*. *(V*.*) panamensis* and *L*. *(V*.*) braziliensis*, the levels of change in the EC_50_ values were 6.95 and 1.49, respectively.

#### *In vitro* susceptibility of intracellular amastigotes of the clinical isolates to AmB

**Fig 3:**
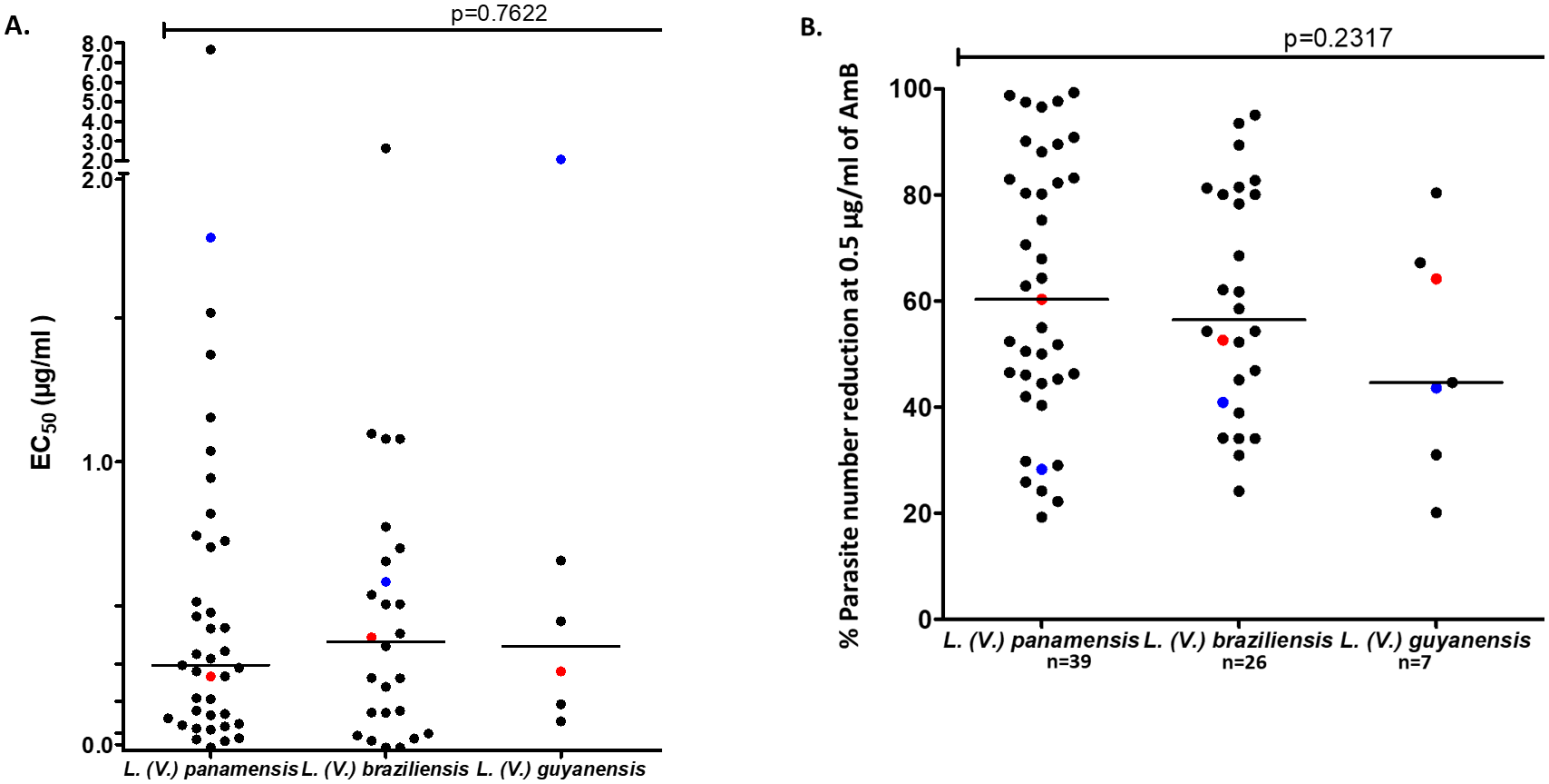
*In vitro* susceptibility of clinical isolates of *Leishmania Viannia* to AmB by species. A) Distribution of the mean effective concentration (EC_50_) in μg/ml AmB for the strains according to species. B) Distribution of the percent reduction in the number of parasites at a concentration of 0.5 μg/ml AmB for the strains according to species.

To establish the baseline susceptibility to AmB of the main *Leishmania* species of subgenus *Viannia*, the EC_50_ values were determined for 65 clinical isolates. Variability was found in the EC_50_ values for the clinical isolates of different species, as shown in Fig 3A. For *L*. *(V*.*) panamensis*, the median EC_50_ value for AmB was 0.295 ± 1.238 μg/ml, with a range of 0.01 to 7.67 μg/ml. For isolates of *L*. *(V*.*) braziliensis*, the median EC_50_ was 0.3763 ± 0.5551 μg/ml, and the mean effective concentration ranged between 0.01 and 2.639 μg/ml.

The EC_50_ value for the strains of the species *L*. *(V*.*) guyanensis* had a mean value that was very close to that obtained for the species *L*. *(V*.*) braziliensis*, with a mean of 0.361 ± 0.7418 μg/ml AmB and a range of variation between 0.101 and 2.076 μg/ml (Fig 3A). Generally, the broadest range of EC_50_ values in the strains was obtained for the species *L*. *(V*.*) panamensis*, whereas the smallest range of variation was obtained for the species *L*. *(V*.*) guyanensis*, which might be due to the small sample size. The percentage reduction in the number of parasites in the presence of 0.5 μg/ml AmB was calculated by species and is shown in Fig 3B.

Half of the strains of the *L*. *(V*.*) panamensis* species had parasite loads that were reduced by at least 60.33%. The minimum and maximum reductions of these strains ranged between 19.23% and 99.32%. Half of the strains of the species *L*. *(V*.*) braziliensis* showed a reduction in the parasite count of 56.44%, with minimum and maximum reductions of 24.17% and 95.04%, respectively. The median percent reduction in the strains of the species *L*. *(V*.*) guyanensis* was 44.64%, but this measure presented a wide range of variation between 20.14% and 80.38%.

The results show great intraspecies variability in the EC_50_ values and percent reductions; however no significant differences were found among the medians of the EC_50_ values (p = 0.762) nor among the percent reductions among the three species evaluated (p = 0.2317). These results suggest that clinical isolates with a lower degree of susceptibility to AmB can be identified within the species. The values recorded for the sensitive control strains were very close to the medians of the isolates for each species, suggesting that this control exhibited representative behavior for the species in terms of susceptibility to AmB.

### Classification of clinical isolates according to the profile of in vitro susceptibility to AmB

The classification dendograms obtained using Ward’s method allowed us to identify groups with common characteristics in the EC_50_ values and percentages of parasite reduction for each species. We identified two groups, sensitive and less sensitive, for *L*. *(V*.*) panamensis* and *L*. *(V*.*) guyanensis*. For *L*. *(V*.*) braziliensis*, three groups were identified: less sensitive, sensitive and more sensitive (Table 1).

**Table 1:** EC_50_ values according to the susceptibility classification.

EC_50_ (μg/ml)	*L*.*(V*.*) panamensis*		*L*. *(V*.*) braziliensis*			*L*. *(V*.*) guyanensis*	
	Less sensitive	Sensitive	Less sensitive	Sensitive	More sensitive	Less sensitive	Sensitive
**N**	11	28	9	8	9	3	3
**Mean**	1.679	0.2163	1.000	0.3289	0.1122	1.0607	0.1783
**Standard deviation**	2.018	0.1526	0.6593	0.2183	0.084	0.8856	0.0879
**Median**	1.038	0.1795	0.775	0.3765	0.13	0.658	0.16
**Rank**	0.705–7.67	0.01–0.515	0.507–2.639	0.01–0.655	0.01–0.25	0.448–2.076	0.101–0.274
**First quartile (25%)**	0.745	0.085	0.584	0.151	0.041	0.448	0.101
**Third quartile (75%)**	1.517	0.326	1.08	0.455	0.137	2.076	0.274

**Fig 4:**
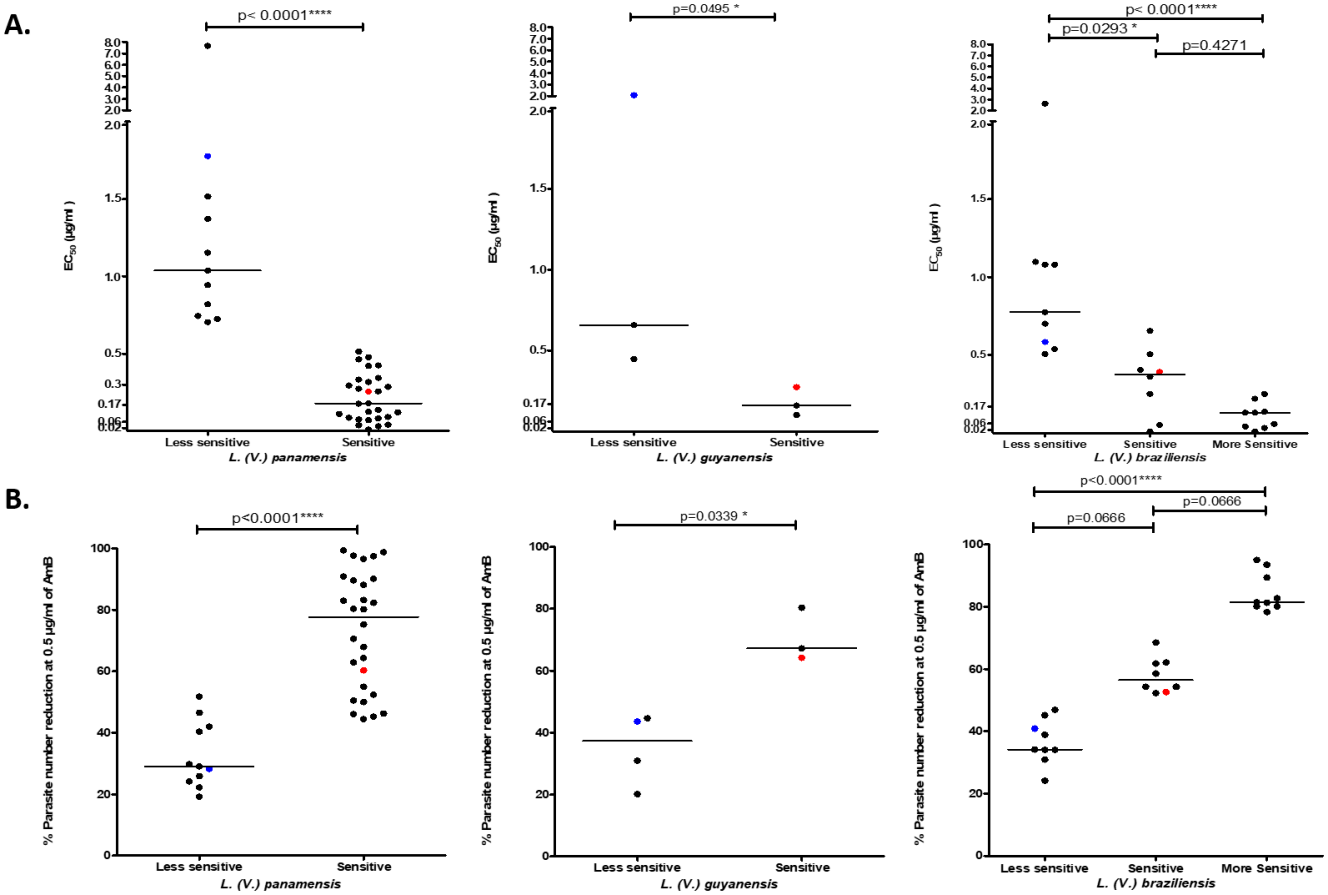
*In vitro* susceptibility to AmB of clinical *Leishmania Viannia* isolates according to the classification of the susceptibility profiles. A) Distribution of EC_50_ values according to the susceptibility classification for each of the species: from left to right, *L*. *(V*.*) panamensis*, *L*. *(V*.*) guyanensis* and *L*. *(V*.*) braziliensis*. B) Distribution of the percentages of parasite reduction according to the classification of susceptibility for each of the species: from left to right, *L*. *(V*.*) panamensis*, *L*. *(V*.*) guyanensis* and *L*. *(V*.*) braziliensis*.

Significant differences were found in the median EC_50_ values of the sensitive and less sensitive strains for *L*. *(V*.*) panamensis* (p<0.0001) and *L*. *(V*.*) guyanensis* (p = 0.0495) (Fig 4A). Regarding *L*. *(V*.*) braziliensis*, the results showed significant differences between the median EC_50_ values in the three susceptibility categories (sensitive, less sensitive and more sensitive) (p = 0.0001). Dunn’s multiple comparisons test showed differences between the median EC_50_ values of the less sensitive strains with respect to the median of the sensitive (p = 0.0293) and more sensitive (p<0.0001) strains, whereas no significant differences were found between the median EC_50_ values of the sensitive and more sensitive strains (p = 0.4271) (Fig 4A).

Significant differences were found between the median percent reductions of the parasites for the sensitive and less sensitive strains for both *L*. *(V*.*) panamensis* (p<0.0001) and *L*. *(V*.*) guyanensis* (p = 0.0339) (Fig 4B). Moreover, differences were found between the median parasite reduction percentages for the three susceptibility categories for strains of the species *L*. *(V*.*) braziliensis* (sensitive, less sensitive and more sensitive) (p<0 0001). Dunn’s multiple comparisons test showed differences only between the median percent reduction of the less sensitive strains compared to the more sensitive strains (p<0.0001).

## Discussion

This study shows for the first time the profile of the *in vitro* susceptibility profile of clinical *L*. *(V*.*) panamensis*, *L*. *(V*.*) braziliensis and L*. *(V*.*) guyanensis* isolates to AmB in the macrophage-amastigote model. The findings reveal no differences in susceptibility to AmB between species; however, we found intraspecies differences in susceptibility that allowed classification of the isolates into groups based on different levels of susceptibility. Additionally, this study is the first report on the *in vitro* generation of strains with decreased susceptibility to AmB through drug selection pressure for species of subgenus *Viannia*. AmB is a second-line drug for the treatment of cutaneous and mucosal leishmaniasis in patients with therapeutic failure or contraindications for the first-line treatments. The species included in this study did not show differences in their EC_50_ values to AmB, with mean values of 0.295, 0.376 and 0.361 μg/ml for *L*. *(V*.*) panamensis*, *L*. *(V*.*) braziliensis* and *L*. *(V*.*) guyanensis*, respectively.

Differences in susceptibility can be a natural or acquired phenotype for microorganisms [6, 7]. Continuous exposure of parasites to medication is one cause of the appearance of a decrease in susceptibility; however, this situation is not the case for AmB, as this drug is not the most commonly used medication in Colombia [42]. A study conducted in immunocompromised patients with treatment failure observed that the inhibitory concentration of AmB *in vitro* increased over the course of successive treatments [43], suggesting changes in susceptibility due to exposure to the drug. The use of AmB in Colombia has been limited by availability of the liposomal formulation, which has recently been introduced in the country. Greater use of this medication should be expected; therefore, determining the baseline susceptibility is important for monitoring changes in the susceptibility patterns [44]. Unlike other medications used to treat leishmaniasis, no controlled clinical trials are available for AmB to evaluate the efficacy of the drug for cutaneous and mucosal leishmaniasis, and even fewer trials have identified the infecting species [8, 9]. Therefore, we could not identify differences in susceptibility between species based on reports in the literature.

At the intraspecies level, the situation is different. Variability was found in the EC_50_ values between isolates of the same species, which were classified into groups according to their susceptibility profiles. A total of 28.21% (11/39) of the *L*. *(V*.*) panamensis* strains, 34.61% (9/26) of the *L*. *(V*.*) braziliensis* strains and 50% (3/6) of the *L*. *(V*.*) guyanensis* strains were classified as less sensitive isolates. The intraspecies variation can be partially explained by the geographical differences of the isolates and the possible existence of subpopulations of the parasite species. Although no work has demonstrated differences in susceptibility to this medication by geographic region in Colombia, the same species from different countries have been reported to require different doses to achieve similar cure rates [45]. For other medications, such as meglumine antimoniate and miltefosine, variability in susceptibility has been reported for the different geographic regions of Colombia, as has the existence of subpopulations of *L*. *(V*.*) panamensis* with different levels of susceptibility to meglumine antimoniate [26].

The natural susceptibility of some of the isolates included in the study exceeded the maximum concentration of free AmB in the plasma [34]. Although the *in vitro* susceptibility failed to reproduce the conditions of an *in vivo* system, this finding suggested that higher concentrations of AmB might be needed in the future to eliminate the parasite. In the reviewed literature, only three studies were found that reported the *in vitro* susceptibility of clinical isolates of the species of subgenus *Viannia* to AmB; however, these studies used parasites in the promastigote stage, which was in contrast to the present study. In 2010, Zauli-Nascimento evaluated the susceptibility of ten clinical isolates of *L*. *(V*.*) braziliensis* from Brazil and reported EC_50_ values in the range of 0.036 to 0.092 μg/ml AmB following 24 hours of exposure to the medication [46]. In 2015, Salamanca evaluated 16 *L*. *(V*.*) braziliensis* isolates from Bolivia and described EC_50_ values that oscillated between 0.03 and 0.41 μg/ml with 72 hours of exposure time [47]. The third study was conducted by Ginouves and collaborators in 2017 with 33 *L*. *(V*.*) guyanensis* and two *L*. *(V*.*) braziliensis* isolates from French Guiana [24]. The authors reported EC_50_ values with wide variability, ranging between 1.03 and >25 μg/ml AmB for *L*. *(V*.*) guyanensis* and between >0.78 and 1.27 μg/ml for *L*. *(V*.*) braziliensis*. The variation in EC_50_ results between studies could be associated with the experimental conditions of each study, such as the parasite concentration, growth phase used and drug concentration [48]. Other variables to consider are the time of exposure to the drug [49, 50], the culture medium used [24, 51], the formulation and the method used to assess the effect of the medication [35]. Differences in these variables between experiments make comparisons of the results difficult. The EC_50_ values obtained in the present work for *L*. *(V*.*) braziliensis* present a range of susceptibility that is much wider than previous reports [24, 46, 47]. The reverse was found for *L*. *(V*.*) guyanensis*. This pattern could be partially explained by the differences in the numbers of isolates in the two studies and the prevalence of this species in Colombia and French Guiana [24]. No reports were found for clinical isolates of *L*. *(V*.*) panamensis*.

The susceptibility of *L*. *(V) panamensis* to AmB has only been reported for reference strains. In one of the few studies conducted by Escobar and collaborators in 2002, these authors reported EC_50_ values of 0.034 μg/ml for promastigotes and 0.073 μg/ml for intracellular amastigotes [19]. In 2009, Varela and colleagues reported an EC_50_ value of 0.078 μg/ml for intracellular amastigotes of a *L*. *(V*.*) panamensis* isolate from a Colombian patient that was transfected with GFP [20]. For the isolates evaluated in the present work, sensitive strains were found to exhibit an average EC_50_ value of 0.2163 μg/ml and less sensitive strains an EC_50_ value of 1.679 μg/ml AmB, which differed from the reports on reference strains. No reports have evaluated the intraspecies variability of the susceptibility profiles to AmB.

In addition to evaluating clinical isolates of the species, the present work also included internal standards of sensitive strains (reference strains) and less sensitive strains generated in the laboratory through selection by increasing the concentrations of the drug, as has been reported for other species [28, 52]. The results obtained in this work constitute the first report of a reduction in the *in vitro* susceptibility to AmB of species of subgenus *Viannia*. The isolates and strains generated in the laboratory that were less sensitive to AmB should be studied in future works to describe the genetic or biochemical mechanisms that confer this phenotype. The characterization of clinical isolates from patients with little response to AmB has been reported mainly for the *L*. *(L*.*) donovani* species in the Bihar region of India [15, 29, 53, 54]. Alterations in the sterol composition of the membrane [53] and alterations in the expression levels of enzymes of the thiol cascade, such as trypanothione reductase [55] and tripareduccin [15], have been reported for these isolates, among other mechanisms [54]. Changes have also been reported in the levels of other enzymes related to the control of reactive oxygen species, such as ascorbate peroxidase [56], cysteine synthetase [53] and cysteine proteinase B [54]. Finally, other more general mechanisms have been described, such as the expression of drug transporters encoded by the MDR1 gene family [54, 57]. These types of alterations may be identified in the isolates less sensitive to AmB found in this work in future studies.

Although the results of the present work suggest variability in the *in vitro* susceptibility and the circulation of less sensitive strains for each species, there have been no reported cases of AmB therapeutic failure in the Americas. However, there are reports of relapse cases after completion of the treatment scheme [11,13] because therapeutic failure is a multifactorial phenomenon that is affected by the characteristics of the parasite, the host and the medicine. Therefore, the reduction in susceptibility levels alone is not a a determining factor for the occurrence of therapeutic failure [58].

This study established the baseline susceptibility to AmB in isolates of subgenus *Viannia* in Colombia. However, given the lack of AmB-controlled clinical trials for the clinical forms of cutaneous and mucosal leishmaniasis, we cannot establish a correlation between *in vitro* test results and the clinical response. Future controlled clinical studies are necessary to establish the effectiveness of the medication. The results found in this work constitute a starting point for monitoring the *in vitro* susceptibility of circulating species in Colombia to AmB, which will result in a better use of this therapeutic agent and extend its life. These findings could impact the health system by reducing the need for retreatments, avoiding relapses and reducing costs, which contribute to prolonged disability of the patient.

## Supporting information

S1 Table Parasites number quantification after AmB exposure. (PDF)

S1 Fig Dendrogram of the susceptibility classification of Leishmania strains obtained using the EC50 values and percent reductions. The clinical isolates were classified into different groups according to the degree of *in vitro* susceptibility to AmB using Ward’s method of hierarchical classification and the EC50 values and percent reductions. A) *L*. *(V*.*) panamensis*, B). *L*. *(V*.*) braziliensis* C) *L*. *(V*.*) guyanensis*.
(PDF)
