# Supplementary material for: Intraspecies differences in natural susceptibility to amphotericine B of clinical isolates of Leishmania subgenus Viannia
Source: PLoS One. 2018 Apr 26;13(4):e0196247. doi: 10.1371/journal.pone.0196247 (PMC5919572; doi:10.1371/journal.pone.0196247)
Supplement: S1 Table — (PDF) [file pone.0196247.s001.pdf]

S1 table. Parasites number quantification after AmB exposure

Strain	Specie	AmB (µg/ml)	Parasite number in experiment 1	Parasite number in experiment 2	Parasite number in experiment 3	mean
Leishmania panamensis Sensitive	*L. (V.) panamensis*	0.500	1.77E+07	1.10E+07	1.47E+07	1.45E+07
		0.170	7.44E+06	9.46E+06	4.60E+07	2.10E+07
		0.060	2.33E+07	2.39E+07	2.78E+07	2.50E+07
		0.020	2.08E+07	3.18E+07	2.81E+07	2.69E+07
		0.000	3.20E+07	3.90E+07	3.84E+07	3.65E+07
Leishmania panamensis Less sensitive	*L. (V.) panamensis*	0.500	4.59E+07	3.46E+07	6.20E+07	4.75E+07
		0.170	5.76E+07	4.20E+07	5.30E+07	5.09E+07
		0.060	6.20E+07	6.64E+07	5.27E+07	6.04E+07
		0.020	6.37E+07	6.84E+07	5.96E+07	6.39E+07
		0.000	7.76E+07	6.80E+07	5.29E+07	6.62E+07
Leishmania braziliensis Sensitive	*L. (V.) braziliensis*	0.500	2.28E+07	2.55E+07	2.07E+07	2.30E+07
		0.170	4.14E+07	2.89E+07	2.25E+07	3.09E+07
		0.060	3.69E+07	5.00E+07	3.31E+07	4.00E+07
		0.020	4.56E+07	5.31E+07	3.98E+07	4.62E+07
		0.000	4.56E+07	5.98E+07	4.03E+07	4.85E+07
Leishmania braziliensis Less sensitive	*L. (V.) braziliensis*	0.500	4.07E+07	2.92E+07	4.98E+07	3.99E+07
		0.170	5.15E+07	4.21E+07	5.32E+07	4.89E+07
		0.060	7.12E+07	5.72E+07	6.53E+07	6.46E+07
		0.020	5.28E+07	6.71E+07	8.22E+07	6.74E+07
		0.000	8.06E+07	7.01E+07	5.17E+07	6.75E+07
Leishmania guyanensis Sensitive	*L. (V.) guyanensis*	0.500	1.92E+06	4.94E+06	5.86E+06	4.24E+06
		0.170	1.17E+07	2.87E+06	9.40E+06	7.98E+06
		0.060	8.62E+06	7.98E+06	8.46E+06	8.35E+06
		0.020	8.86E+06	1.21E+07	1.31E+07	1.13E+07
		0.000	1.36E+07	9.36E+06	1.26E+07	1.18E+07
LL010	*L. (V.) braziliensis*	0.500	7.44E+06	5.76E+06	8.17E+06	7.12E+06
		0.170	6.93E+06	9.09E+06	9.60E+06	8.54E+06
		0.060	9.22E+06	9.26E+06	9.92E+06	9.46E+06
		0.020	1.17E+07	1.01E+07	1.02E+07	1.07E+07
		0.000	1.10E+07	8.62E+06	1.28E+07	1.08E+07
LL014	*L. (V.) braziliensis*	0.500	2.27E+06	2.99E+06	2.78E+06	2.68E+06
		0.170	8.68E+06	7.94E+06	8.90E+06	8.50E+06
		0.060	1.20E+07	1.11E+07	1.41E+07	1.24E+07
		0.020	1.17E+07	1.31E+07	9.09E+06	1.13E+07
		0.000	1.31E+07	1.12E+07	1.28E+07	1.24E+07
LL027	*L. (V.) braziliensis*	0.500	9.94E+05	8.36E+05	1.10E+06	9.77E+05
		0.170	6.20E+05	6.29E+05	6.44E+05	6.31E+05
		0.060	1.07E+06	9.01E+05	1.01E+06	9.95E+05

		0.020	2.80E+06	2.30E+06	2.14E+06	2.41E+06
		0.000	1.15E+06	2.02E+06	1.63E+06	1.60E+06
LL033	*L. (V.) braziliensis*	0.500	6.04E+06	6.12E+06	6.56E+06	6.24E+06
		0.170	7.25E+06	6.62E+06	9.25E+06	7.71E+06
		0.060	8.23E+06	7.22E+06	5.27E+06	6.91E+06
		0.020	1.26E+07	1.23E+07	9.83E+06	1.16E+07
		0.000	8.20E+06	1.01E+07	8.78E+06	9.04E+06
LL035	*L. (V.) braziliensis*	0.500	2.01E+06	9.38E+05	1.25E+06	1.40E+06
		0.170	1.96E+06	2.07E+06	1.92E+06	1.98E+06
		0.060	2.51E+06	3.18E+06	2.84E+06	2.85E+06
		0.020	3.53E+06	2.47E+06	2.20E+06	2.73E+06
		0.000	3.84E+06	3.72E+06	3.41E+06	3.66E+06
LL036	*L. (V.) braziliensis*	0.500	3.22E+06	4.00E+06	2.98E+06	3.40E+06
		0.170	4.28E+06	4.87E+06	3.80E+06	4.32E+06
		0.060	2.45E+06	2.29E+06	2.64E+06	2.46E+06
		0.020	6.51E+06	6.16E+06	5.76E+06	6.14E+06
		0.000	8.77E+06	1.03E+07	7.85E+06	8.98E+06
LL223	*L. (V.) braziliensis*	0.500	3.77E+06	3.87E+06	3.99E+06	3.88E+06
		0.170	9.69E+06	7.89E+06	6.79E+06	8.13E+06
		0.060	8.25E+06	6.01E+06	7.12E+06	7.13E+06
		0.020	9.02E+06	8.85E+06	1.12E+07	9.69E+06
		0.000	9.89E+06	8.37E+06	9.81E+06	9.35E+06
LL051	*L. (V.) panamensis*	0.500	2.93E+07	3.26E+07	3.09E+07	3.09E+07
		0.170	2.93E+07	3.72E+07	2.83E+07	3.16E+07
		0.060	3.27E+07	4.15E+07	3.49E+07	3.64E+07
		0.020	5.22E+07	5.51E+07	5.01E+07	5.25E+07
		0.000	5.87E+07	5.27E+07	5.57E+07	5.57E+07
LL045	*L. (V.) panamensis*	0.500	2.31E+07	2.64E+07	2.48E+07	2.48E+07
		0.170	2.31E+07	3.11E+07	2.21E+07	2.54E+07
		0.060	2.65E+07	3.53E+07	2.88E+07	3.02E+07
		0.020	4.60E+07	4.89E+07	4.39E+07	4.63E+07
		0.000	5.25E+07	4.65E+07	4.95E+07	4.95E+07
LL055	*L. (V.) panamensis*	0.500	1.96E+07	1.84E+07	2.70E+07	2.17E+07
		0.170	2.05E+07	2.39E+07	2.39E+07	2.28E+07
		0.060	3.07E+07	3.37E+07	3.03E+07	3.16E+07
		0.020	2.77E+07	4.33E+07	3.92E+07	3.68E+07
		0.000	3.50E+07	4.24E+07	4.13E+07	3.96E+07
LL058	*L. (V.) panamensis*	0.500	2.69E+07	3.11E+07	2.66E+07	2.82E+07
		0.170	3.67E+07	3.49E+07	2.84E+07	3.33E+07
		0.060	2.89E+07	3.23E+07	3.61E+07	3.24E+07
		0.020	4.63E+07	3.82E+07	4.08E+07	4.17E+07
		0.000	5.27E+07	5.34E+07	5.20E+07	5.27E+07
LL072	*L. (V.) panamensis*	0.500	3.10E+07	3.02E+07	3.17E+07	3.10E+07
		0.170	3.85E+07	3.14E+07	3.64E+07	3.55E+07

		0.060	3.37E+07	2.97E+07	4.70E+07	3.68E+07
		0.020	2.99E+07	5.31E+07	5.13E+07	4.48E+07
		0.000	4.81E+07	3.96E+07	3.48E+07	4.08E+07
LL074	*L. (V.) panamensis*	0.500	4.38E+07	4.41E+07	4.38E+07	4.39E+07
		0.170	5.72E+07	5.70E+07	6.05E+07	5.82E+07
		0.060	6.64E+07	7.99E+07	6.60E+07	7.08E+07
		0.020	9.17E+07	1.18E+08	1.00E+08	1.03E+08
		0.000	7.48E+07	9.07E+07	1.01E+08	8.87E+07
LL078	*L. (V.) guyanensis*	0.500	1.28E+07	3.60E+06	9.73E+06	8.73E+06
		0.170	1.13E+07	1.23E+07	1.17E+07	1.17E+07
		0.060	1.50E+07	9.90E+06	1.29E+07	1.26E+07
		0.020	1.17E+07	9.72E+06	1.19E+07	1.11E+07
		0.000	1.25E+07	8.52E+06	1.17E+07	1.09E+07
LL086	*L. (V.) braziliensis*	0.500	2.46E+05	2.53E+05	2.03E+05	2.34E+05
		0.170	9.92E+05	9.92E+05	8.95E+05	9.59E+05
		0.060	5.99E+05	4.65E+05	3.38E+05	4.67E+05
		0.020	3.54E+06	3.62E+06	3.78E+06	3.65E+06
		0.000	5.73E+06	3.87E+06	4.56E+06	4.72E+06
LL106	*L. (V.) panamensis*	0.500	6.58E+06	6.14E+06	5.94E+06	6.22E+06
		0.170	2.36E+07	3.20E+07	2.49E+07	2.68E+07
		0.060	1.88E+07	2.69E+07	3.05E+07	2.54E+07
		0.020	3.05E+07	2.85E+07	3.22E+07	3.04E+07
		0.000	3.31E+07	2.90E+07	3.27E+07	3.16E+07
LL107	*L. (V.) panamensis*	0.500	2.85E+07	2.66E+07	2.31E+07	2.61E+07
		0.170	2.84E+07	2.74E+07	2.90E+07	2.83E+07
		0.060	3.00E+07	2.82E+07	2.74E+07	2.85E+07
		0.020	3.03E+07	3.72E+07	3.09E+07	3.28E+07
		0.000	3.39E+07	3.39E+07	3.77E+07	3.52E+07
LL110	*L. (V.) braziliensis*	0.500	6.11E+04	9.05E+04	6.88E+04	7.34E+04
		0.170	1.14E+05	1.16E+05	8.02E+04	1.03E+05
		0.060	1.56E+05	1.30E+05	1.49E+05	1.45E+05
		0.020	1.54E+05	1.77E+05	1.13E+05	1.48E+05
		0.000	1.52E+05	1.53E+05	1.78E+05	1.61E+05
LL117	*L. (V.) panamensis*	0.500	2.02E+07	1.75E+07	2.03E+07	1.93E+07
		0.170	1.24E+07	2.60E+07	2.51E+07	2.12E+07
		0.060	2.73E+07	2.81E+07	2.77E+07	2.77E+07
		0.020	3.34E+07	4.36E+07	3.54E+07	3.75E+07
		0.000	5.53E+07	7.95E+07	6.28E+07	6.59E+07
LL113	*L. (V.) panamensis*	0.500	2.32E+07	2.34E+07	2.58E+07	2.41E+07
		0.170	2.38E+07	2.80E+07	2.48E+07	2.55E+07
		0.060	2.54E+07	2.79E+07	4.12E+07	3.15E+07
		0.020	3.24E+07	3.95E+07	2.76E+07	3.32E+07
		0.000	4.41E+07	3.19E+07	2.61E+07	3.40E+07
LL119	*L. (V.) panamensis*	0.500	5.41E+06	5.91E+06	5.53E+06	5.62E+06

		0.170	1.87E+07	1.59E+07	1.42E+07	1.63E+07
		0.060	2.68E+07	2.33E+07	2.74E+07	2.59E+07
		0.020	2.66E+07	1.81E+07	2.72E+07	2.39E+07
		0.000	2.90E+07	3.17E+07	3.96E+07	3.35E+07
LL123	*L. (V.) panamensis*	0.500	1.95E+07	1.04E+07	1.50E+07	1.49E+07
		0.170	2.09E+07	2.34E+07	2.25E+07	2.23E+07
		0.060	2.06E+07	2.86E+07	1.81E+07	2.24E+07
		0.020	3.94E+07	3.34E+07	3.94E+07	3.74E+07
		0.000	2.46E+07	2.73E+07	2.53E+07	2.57E+07
LL125	*L. (V.) panamensis*	0.500	3.23E+07	4.89E+07	4.58E+07	4.23E+07
		0.170	4.38E+07	3.99E+07	4.46E+07	4.28E+07
		0.060	4.77E+07	4.72E+07	4.41E+07	4.63E+07
		0.020	4.89E+07	5.02E+07	6.23E+07	5.38E+07
		0.000	5.12E+07	5.42E+07	5.17E+07	5.24E+07
LL152	*L. (V.) panamensis*	0.500	2.27E+07	1.82E+07	2.09E+07	2.06E+07
		0.170	4.18E+07	3.96E+07	2.67E+07	3.60E+07
		0.060	3.18E+07	3.68E+07	3.40E+07	3.42E+07
		0.020	2.99E+07	4.16E+07	3.20E+07	3.45E+07
		0.000	5.08E+07	3.39E+07	4.35E+07	4.27E+07
LL126	*L. (V.) panamensis*	0.500	1.03E+07	9.92E+06	1.22E+07	1.08E+07
		0.170	1.06E+07	1.29E+07	1.17E+07	1.18E+07
		0.060	1.10E+07	1.18E+07	1.43E+07	1.24E+07
		0.020	1.78E+07	1.59E+07	1.65E+07	1.67E+07
		0.000	1.98E+07	1.82E+07	1.63E+07	1.81E+07
LL129	*L. (V.) panamensis*	0.500	2.20E+07	2.41E+07	3.10E+07	2.57E+07
		0.170	3.99E+07	3.49E+07	4.27E+07	3.92E+07
		0.060	4.51E+07	3.79E+07	3.67E+07	3.99E+07
		0.020	8.59E+07	7.51E+07	8.50E+07	8.20E+07
		0.000	1.04E+08	1.18E+08	9.05E+07	1.04E+08
LL131	*L. (V.) panamensis*	0.500	1.09E+07	1.21E+07	1.35E+07	1.21E+07
		0.170	1.73E+07	1.88E+07	1.73E+07	1.78E+07
		0.060	1.33E+07	2.04E+07	2.24E+07	1.87E+07
		0.020	2.17E+07	2.22E+07	1.72E+07	2.04E+07
		0.000	2.44E+07	1.99E+07	2.32E+07	2.25E+07
LL138	*L. (V.) panamensis*	0.500	8.14E+05	7.78E+04	8.25E+05	5.72E+05
		0.170	9.89E+05	8.57E+05	8.66E+05	9.04E+05
		0.060	7.17E+06	7.51E+06	1.01E+07	8.25E+06
		0.020	6.99E+06	7.73E+06	5.16E+06	6.63E+06
		0.000	2.59E+07	2.59E+07	1.69E+07	2.29E+07
LL186	*L. (V.) panamensis*	0.500	1.16E+07	1.10E+07	1.10E+07	1.12E+07
		0.170	9.94E+06	1.60E+07	1.47E+07	1.35E+07
		0.060	1.58E+07	1.17E+07	1.74E+07	1.49E+07
		0.020	1.77E+07	1.54E+07	1.72E+07	1.68E+07
		0.000	1.59E+07	1.76E+07	1.43E+07	1.59E+07

LL142	*L. (V.) panamensis*	0.500	9.04E+06	1.16E+07	6.40E+06	9.00E+06
		0.170	1.87E+07	1.29E+07	1.86E+07	1.67E+07
		0.060	1.62E+07	2.12E+07	1.81E+07	1.85E+07
		0.020	1.40E+07	1.87E+07	1.58E+07	1.62E+07
		0.000	2.77E+07	2.16E+07	2.63E+07	2.52E+07
LL277	*L. (V.) braziliensis*	0.500	7.14E+06	9.27E+06	9.70E+06	8.70E+06
		0.170	2.60E+07	2.47E+07	2.10E+07	2.39E+07
		0.060	2.04E+07	2.03E+07	3.37E+07	2.48E+07
		0.020	4.67E+07	4.62E+07	4.45E+07	4.58E+07
		0.000	4.14E+07	5.49E+07	4.46E+07	4.70E+07
LL282	*L. (V.) braziliensis*	0.500	3.25E+05	2.96E+05	2.01E+05	2.74E+05
		0.170	2.03E+06	1.77E+06	2.30E+06	2.03E+06
		0.060	3.01E+06	6.27E+06	4.23E+06	4.50E+06
		0.020	5.86E+06	2.92E+06	3.33E+06	4.03E+06
		0.000	3.60E+06	5.42E+06	3.64E+06	4.22E+06
LL298	*L. (V.) braziliensis*	0.500	1.90E+06	1.84E+06	5.67E+05	1.44E+06
		0.170	1.44E+06	1.73E+06	1.66E+06	1.61E+06
		0.060	3.53E+06	2.99E+06	2.55E+06	3.02E+06
		0.020	9.60E+06	1.12E+07	1.24E+07	1.11E+07
		0.000	1.67E+07	1.04E+07	1.35E+07	1.35E+07
LL320	*L. (V.) braziliensis*	0.500	9.26E+06	1.53E+07	1.62E+07	1.36E+07
		0.170	1.72E+07	1.27E+07	1.27E+07	1.42E+07
		0.060	2.80E+06	2.39E+07	2.36E+07	1.67E+07
		0.020	2.09E+07	2.80E+07	1.78E+07	2.23E+07
		0.000	2.39E+07	2.05E+07	1.76E+07	2.07E+07
LL104	*L. (V.) braziliensis*	0.500	4.25E+06	4.01E+06	5.61E+06	4.62E+06
		0.170	6.10E+06	4.49E+06	6.73E+06	5.77E+06
		0.060	5.71E+06	6.10E+06	6.50E+06	6.10E+06
		0.020	6.78E+06	6.46E+06	6.36E+06	6.53E+06
		0.000	4.95E+06	7.22E+06	6.12E+06	6.10E+06
LL321	*L. (V.) panamensis*	0.500	1.20E+07	6.44E+06	1.24E+07	1.03E+07
		0.170	1.26E+07	2.41E+07	1.27E+07	1.65E+07
		0.060	2.31E+07	2.50E+07	1.88E+07	2.23E+07
		0.020	2.86E+07	2.16E+07	2.74E+07	2.59E+07
		0.000	2.76E+07	2.77E+07	4.08E+07	3.20E+07
LL299	*L. (V.) panamensis*	0.500	1.24E+07	1.26E+07	7.75E+06	1.09E+07
		0.170	1.12E+07	9.82E+06	1.09E+07	1.06E+07
		0.060	1.29E+07	1.36E+07	1.62E+07	1.42E+07
		0.020	1.82E+07	2.18E+07	2.07E+07	2.02E+07
		0.000	1.92E+07	2.12E+07	2.06E+07	2.03E+07
LL530	*L. (V.) panamensis*	0.500	7.14E+06	5.97E+06	1.22E+06	4.78E+06
		0.170	3.77E+06	4.08E+06	4.39E+06	4.08E+06
		0.060	1.29E+07	1.08E+07	9.21E+06	1.10E+07
		0.020	1.29E+07	1.21E+07	8.48E+06	1.12E+07

		0.000	1.28E+07	1.36E+07	1.22E+07	1.29E+07
LL309	*L. (V.) braziliensis*	0.500	1.75E+06	8.88E+05	8.15E+05	1.15E+06
		0.170	2.59E+06	3.46E+06	3.82E+06	3.29E+06
		0.060	3.26E+06	4.32E+06	3.01E+06	3.53E+06
		0.020	5.03E+06	5.56E+06	3.27E+06	4.62E+06
		0.000	4.81E+06	6.30E+06	6.24E+06	5.78E+06
LL315	*L. (V.) braziliensis*	0.500	3.42E+06	2.32E+06	6.77E+06	4.17E+06
		0.170	5.33E+06	5.58E+06	2.65E+06	4.52E+06
		0.060	1.30E+07	9.89E+06	1.25E+07	1.18E+07
		0.020	2.01E+07	1.85E+07	8.95E+06	1.58E+07
		0.000	1.92E+07	2.38E+07	2.38E+07	2.23E+07
LL324	*L. (V.) panamensis*	0.500	1.03E+06	3.50E+06	1.69E+06	2.07E+06
		0.170	1.09E+07	1.15E+07	1.29E+07	1.17E+07
		0.060	1.08E+07	1.32E+07	1.05E+07	1.15E+07
		0.020	2.43E+07	1.77E+07	1.75E+07	1.98E+07
		0.000	1.87E+07	2.29E+07	1.79E+07	1.99E+07
LL329	*L. (V.) braziliensis*	0.500	1.31E+07	1.31E+07	1.27E+07	1.30E+07
		0.170	1.58E+07	1.31E+07	1.49E+07	1.46E+07
		0.060	1.98E+07	1.74E+07	1.91E+07	1.88E+07
		0.020	2.36E+07	1.71E+07	1.83E+07	1.97E+07
		0.000	2.59E+07	3.03E+07	2.52E+07	2.72E+07
LL334	*L. (V.) panamensis*	0.500	4.03E+06	5.80E+06	9.16E+06	6.33E+06
		0.170	2.02E+07	2.90E+07	2.58E+07	2.50E+07
		0.060	5.71E+07	4.70E+07	6.88E+07	5.77E+07
		0.020	6.52E+07	3.63E+07	5.73E+07	5.29E+07
		0.000	6.35E+07	5.22E+07	7.65E+07	6.41E+07
LL335	*L. (V.) braziliensis*	0.500	1.57E+06	2.61E+06	1.42E+06	1.87E+06
		0.170	5.34E+06	3.12E+06	3.12E+06	3.86E+06
		0.060	9.49E+06	1.07E+07	9.32E+06	9.85E+06
		0.020	1.05E+07	1.20E+07	7.97E+06	1.01E+07
		0.000	9.21E+06	1.08E+07	8.14E+06	9.37E+06
LL114	*L. (V.) braziliensis*	0.500	7.80E+06	6.83E+06	5.55E+06	6.73E+06
		0.170	7.16E+06	6.58E+06	8.73E+06	7.49E+06
		0.060	1.10E+07	1.00E+07	8.06E+06	9.70E+06
		0.020	1.20E+07	1.40E+07	1.24E+07	1.28E+07
		0.000	1.17E+07	1.29E+07	1.34E+07	1.27E+07
LL386	*L. (V.) panamensis*	0.500	3.07E+07	3.16E+07	3.06E+07	3.10E+07
		0.170	4.82E+07	3.59E+07	4.28E+07	4.23E+07
		0.060	4.53E+07	5.06E+07	5.00E+07	4.86E+07
		0.020	6.43E+07	4.92E+07	3.56E+07	4.97E+07
		0.000	6.31E+07	7.85E+07	6.48E+07	6.88E+07
LL522	*L. (V.) panamensis*	0.500	5.18E+06	5.62E+06	5.73E+06	5.51E+06
		0.170	1.90E+07	1.70E+07	1.85E+07	1.82E+07
		0.060	4.27E+07	4.65E+07	5.63E+07	4.85E+07

		0.020	4.64E+07	3.88E+07	5.01E+07	4.51E+07
		0.000	6.20E+07	6.31E+07	5.58E+07	6.03E+07
LL574	*L. (V.) panamensis*	0.500	1.18E+06	1.31E+06	1.57E+06	1.35E+06
		0.170	2.28E+07	1.21E+07	2.27E+07	1.92E+07
		0.060	2.85E+07	2.64E+07	2.00E+07	2.50E+07
		0.020	4.12E+07	3.62E+07	3.41E+07	3.72E+07
		0.000	3.58E+07	4.07E+07	4.15E+07	3.93E+07
LL578	*L. (V.) panamensis*	0.500	5.30E+05	5.06E+05	5.01E+05	5.12E+05
		0.170	3.81E+07	3.16E+07	3.58E+07	3.52E+07
		0.060	3.05E+07	3.48E+07	4.05E+07	3.53E+07
		0.020	6.57E+07	7.30E+07	7.49E+07	7.12E+07
		0.000	7.62E+07	7.36E+07	7.77E+07	7.58E+07
LL626	*L. (V.) panamensis*	0.500	3.55E+05	3.55E+05	4.56E+05	3.89E+05
		0.170	4.55E+05	3.55E+05	5.55E+05	4.55E+05
		0.060	3.04E+07	2.86E+07	3.19E+07	3.03E+07
		0.020	4.97E+07	2.17E+07	1.18E+07	2.77E+07
		0.000	3.08E+07	3.34E+07	3.02E+07	3.15E+07
LL633	*L. (V.) panamensis*	0.500	5.49E+06	5.94E+06	5.54E+06	5.65E+06
		0.170	7.55E+06	6.76E+06	7.55E+06	7.29E+06
		0.060	2.12E+07	1.89E+07	1.86E+07	1.96E+07
		0.020	1.55E+07	1.21E+07	1.98E+07	1.58E+07
		0.000	3.61E+07	3.39E+07	2.58E+07	3.19E+07
LL323	*L. (V.) panamensis*	0.500	4.26E+06	5.80E+06	5.82E+06	5.29E+06
		0.170	1.17E+08	7.72E+07	7.82E+07	9.09E+07
		0.060	1.22E+08	1.62E+08	1.54E+08	1.46E+08
		0.020	1.74E+08	1.49E+08	1.68E+08	1.64E+08
		0.000	2.29E+08	2.27E+08	2.23E+08	2.26E+08
LL646	*L. (V.) guyanensis*	0.500	2.21E+06	6.46E+06	3.99E+06	4.22E+06
		0.170	5.32E+06	2.35E+06	5.65E+06	4.44E+06
		0.060	7.73E+06	3.42E+06	7.86E+06	6.34E+06
		0.020	1.19E+07	9.91E+06	1.01E+07	1.07E+07
		0.000	9.34E+06	1.53E+07	1.39E+07	1.29E+07
LL319	*L. (V.) guyanensis*	0.500	8.99E+06	9.63E+06	7.19E+06	8.61E+06
		0.170	6.43E+06	3.82E+06	6.99E+06	5.74E+06
		0.060	1.19E+07	1.38E+07	1.45E+07	1.34E+07
		0.020	1.40E+07	1.30E+07	1.36E+07	1.35E+07
		0.000	1.10E+07	1.10E+07	1.54E+07	1.25E+07
LL660	*L. (V.) braziliensis*	0.500	2.31E+06	3.11E+06	2.03E+06	2.48E+06
		0.170	2.93E+06	1.77E+06	2.24E+06	2.31E+06
		0.060	3.14E+06	3.40E+06	3.88E+06	3.47E+06
		0.020	4.50E+06	2.58E+06	3.71E+06	3.60E+06
		0.000	1.00E+07	6.30E+06	7.31E+06	7.88E+06
LL670	*L. (V.) guyanensis*	0.500	5.24E+06	2.66E+06	4.22E+06	4.04E+06
		0.170	1.24E+07	1.14E+07	1.22E+07	1.20E+07

		0.060	1.70E+07	1.45E+07	1.01E+07	1.39E+07
		0.020	1.88E+07	1.71E+07	2.02E+07	1.87E+07
		0.000	2.65E+07	1.54E+07	1.99E+07	2.06E+07
LL130	*L. (V.) braziliensis*	0.500	1.00E+07	7.75E+06	1.10E+07	9.58E+06
		0.170	9.33E+06	1.22E+07	1.29E+07	1.15E+07
		0.060	1.24E+07	1.25E+07	1.33E+07	1.27E+07
		0.020	1.57E+07	1.36E+07	1.38E+07	1.43E+07
		0.000	1.49E+07	1.16E+07	1.72E+07	1.45E+07
LL331	*L. (V.) braziliensis*	0.500	2.89E+06	3.59E+06	2.68E+06	3.05E+06
		0.170	3.84E+06	4.37E+06	3.41E+06	3.87E+06
		0.060	3.51E+06	3.29E+06	3.79E+06	3.53E+06
		0.020	9.35E+06	8.84E+06	8.26E+06	8.82E+06
		0.000	2.26E+07	1.90E+07	1.14E+07	1.77E+07
LL360	*L. (V.) braziliensis*	0.500	7.55E+04	1.27E+05	1.06E+05	1.03E+05
		0.170	1.60E+05	1.62E+05	1.12E+05	1.45E+05
		0.060	1.18E+05	1.82E+05	1.08E+05	1.36E+05
		0.020	1.16E+05	1.47E+05	1.58E+05	1.40E+05
		0.000	2.12E+05	2.14E+05	2.49E+05	2.25E+05
LL273	*L. (V.) panamensis*	0.500	1.74E+06	1.27E+06	1.63E+06	1.55E+06
		0.170	1.31E+06	1.13E+06	1.35E+06	1.26E+06
		0.060	3.22E+06	3.69E+06	2.07E+06	2.99E+06
		0.020	8.76E+06	1.39E+07	1.01E+07	1.09E+07
		0.000	1.52E+07	1.28E+07	1.09E+07	1.30E+07
LL391	*L. (V.) braziliensis*	0.500	3.88E+06	4.95E+06	4.56E+06	4.46E+06
		0.170	5.56E+06	5.55E+06	5.46E+06	5.53E+06
		0.060	7.21E+06	7.53E+06	5.28E+06	6.67E+06
		0.020	6.18E+06	7.98E+06	5.17E+06	6.44E+06
		0.000	7.51E+06	8.91E+06	7.97E+06	8.13E+06
LL390	*L. (V.) panamensis*	0.500	1.43E+06	1.22E+06	1.16E+06	1.27E+06
		0.170	4.87E+06	3.85E+06	3.53E+06	4.08E+06
		0.060	8.66E+06	9.26E+06	7.57E+06	8.50E+06
		0.020	9.54E+06	8.82E+06	6.47E+06	8.28E+06
		0.000	8.40E+06	7.30E+06	6.61E+06	7.44E+06
LL381	*L. (V.) panamensis*	0.500	2.18E+07	2.57E+07	2.17E+07	2.31E+07
		0.170	2.18E+07	3.49E+07	2.93E+07	2.87E+07
		0.060	2.50E+07	2.97E+07	2.52E+07	2.66E+07
		0.020	4.35E+07	5.49E+07	3.85E+07	4.56E+07
		0.000	4.97E+07	5.23E+07	4.34E+07	4.84E+07
LL590	*L. (V.) panamensis*	0.500	6.22E+06	6.90E+06	5.21E+06	6.11E+06
		0.170	2.23E+07	2.60E+07	2.18E+07	2.34E+07
		0.060	2.78E+07	3.03E+07	2.67E+07	2.83E+07
		0.020	2.88E+07	3.20E+07	2.82E+07	2.97E+07
		0.000	3.13E+07	3.25E+07	2.87E+07	3.08E+07
LL682	*L. (V.) panamensis*	0.500	1.10E+07	1.23E+07	9.61E+06	1.10E+07

		0.170	9.40E+06	1.79E+07	1.29E+07	1.34E+07
		0.060	1.49E+07	1.31E+07	1.52E+07	1.44E+07
		0.020	1.67E+07	1.74E+07	1.51E+07	1.64E+07
		0.000	1.50E+07	1.48E+07	1.25E+07	1.41E+07
LL621	*L. (V.) guyanensis*	0.500	6.40E+06	7.93E+06	5.92E+06	6.75E+06
		0.170	6.01E+06	4.73E+06	5.75E+06	5.50E+06
		0.060	1.11E+07	1.71E+07	1.19E+07	1.34E+07
		0.020	1.31E+07	1.61E+07	1.12E+07	1.34E+07
		0.000	1.03E+07	1.36E+07	1.27E+07	1.22E+07
Leishmania guyanensis Less sensitive	*L. (V.) guyanensis*	0.500	1.93E+07	1.43E+07	1.60E+07	1.65E+07
		0.170	2.18E+07	2.74E+07	2.81E+07	2.58E+07
		0.060	2.59E+07	3.25E+07	2.15E+07	2.66E+07
		0.020	2.74E+07	2.44E+07	2.27E+07	2.49E+07
		0.000	2.84E+07	3.57E+07	2.39E+07	2.93E+07
