# Supplementary material for: Intraspecies differences in natural susceptibility to amphotericine B of clinical isolates of Leishmania subgenus Viannia
Source: PLoS One. 2018 Apr 26;13(4):e0196247. doi: 10.1371/journal.pone.0196247 (PMC5919572; doi:10.1371/journal.pone.0196247)

S1 Fig. Dendrogram of the susceptibility classification of Leishmania strains obtained using the EC50 values and percent reductions. The clinical isolates were classified into different groups according to the degree of *in vitro* susceptibility to AmB using Ward's method of hierarchical classification and the EC50 values and percent reductions. A) *L. (V.) panamensis,* B) *L. (V.) braziliensis* C) *L. (V.) guyanensis.*

A) *L. (V.) panamensis*

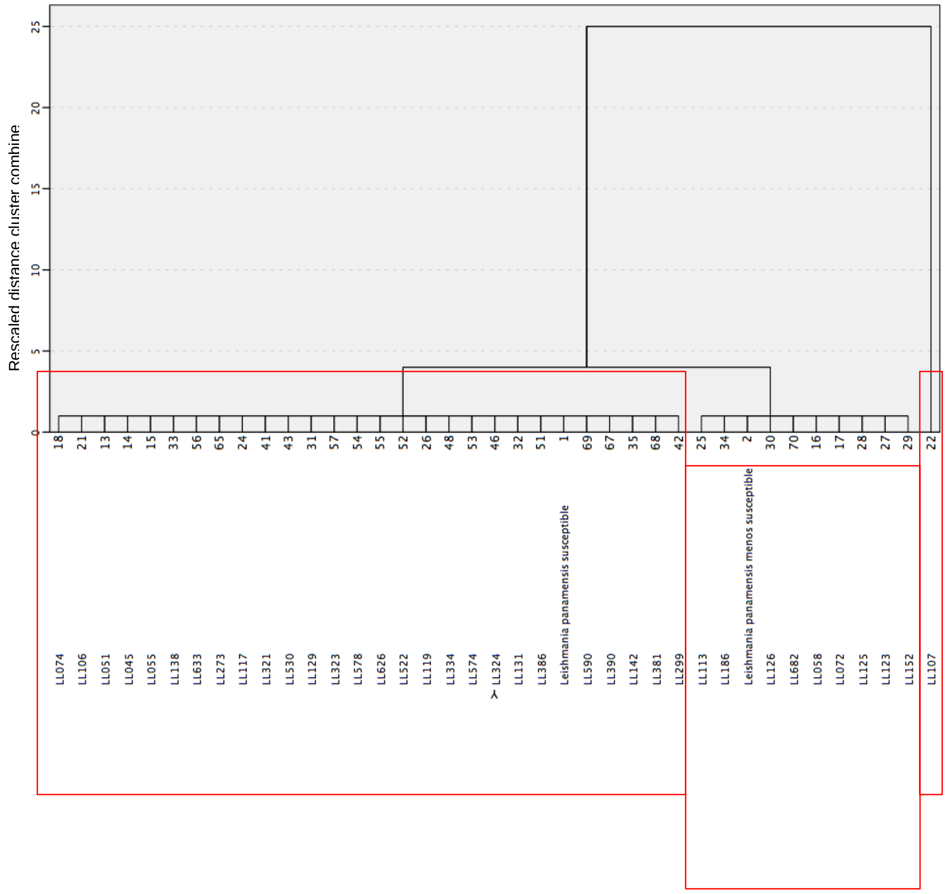

B) *L. (V.) braziliensis*

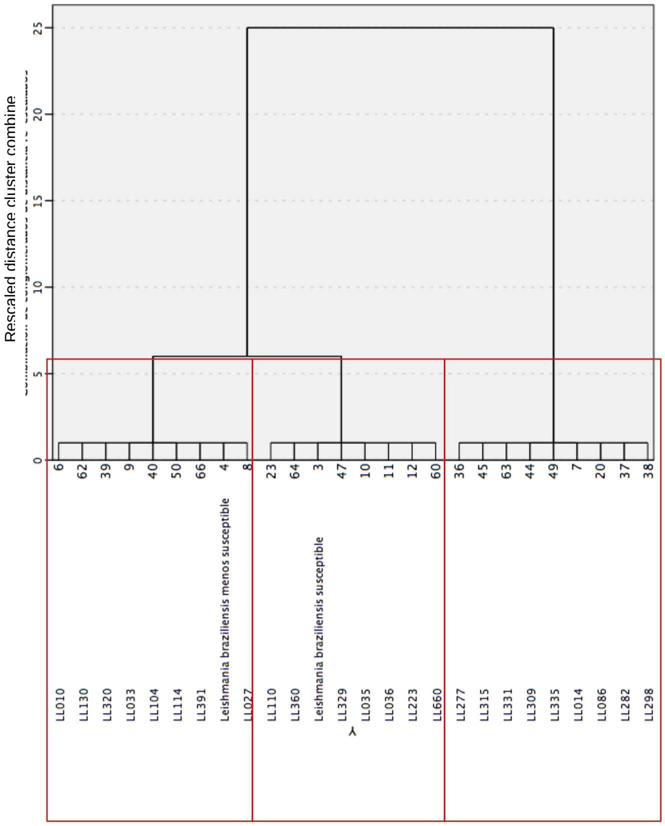

C) *L. (V.) guyanensis*

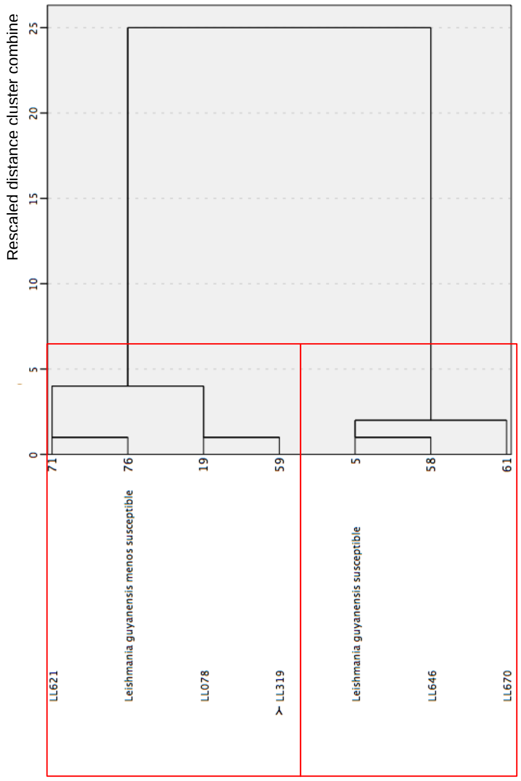

Supplement: S1 Fig — The clinical isolates were classified into different groups according to the degree of in vitro susceptibility to AmB using Ward’s method of hierarchical classification and the EC50 values and percent reductions. A) L. (V.) panamensis, B). L. (V.) braziliensis C) L. (V.) guyanensis. (PDF) [file pone.0196247.s002.pdf]
